# Lessons from IgA Nephropathy Models

**DOI:** 10.3390/ijms252111484

**Published:** 2024-10-25

**Authors:** Toshiki Kano, Hitoshi Suzuki, Yuko Makita, Yoshihito Nihei, Yusuke Fukao, Maiko Nakayama, Mingfeng Lee, Ryosuke Aoki, Koshi Yamada, Masahiro Muto, Yusuke Suzuki

**Affiliations:** 1 Department of Nephrology, Juntendo University Faculty of Medicine, Tokyo 113-8421, Japan; 2 Department of Nephrology, Juntendo University Urayasu Hospital, Chiba 279-0021, Japan

**Keywords:** IgA nephropathy, animal models, galactose deficient IgA1, immune complex, Toll-like receptors, APRIL (a proliferation-inducing ligand), endothelin

## Abstract

IgA nephropathy (IgAN) is the most common type of primary glomerulonephritis worldwide; however, the underlying mechanisms of this disease are not fully understood. This review explores several animal models that provide insights into IgAN pathogenesis, emphasizing the roles of aberrant IgA1 glycosylation and immune complex formation. It discusses spontaneous, immunization, and transgenic models illustrating unique aspects of IgAN development and progression. The animal models, represented by the grouped ddY (gddY) mouse, have provided guidance concerning the multi-hit pathogenesis of IgAN. In this paradigm, genetic and environmental factors, including the dysregulation of the mucosal immune system, lead to increased levels of aberrantly glycosylated IgA, nephritogenic immune complex formation, and subsequent glomerular deposition, followed by mesangial cell activation and injury. Additionally, this review considers the implications of clinical trials targeting molecular pathways influenced by IgAN (e.g., a proliferation-inducing ligand [APRIL]). Collectively, these animal models have expanded the understanding of IgAN pathogenesis while facilitating the development of therapeutic strategies that are currently under clinical investigation. Animal-model-based studies have the potential to facilitate the development of targeted therapies with reduced side effects for IgAN patients.

## 1. Introduction

IgA nephropathy (IgAN) is associated with substantial morbidity, culminating in end-stage renal disease in approximately 30–40% of patients within 20 years after onset [1]. Because of the relatively young age at onset and high frequency of IgAN, it is challenging to elucidate the mechanisms that underlie the disease onset, progression, and inhibition. Aberrant glycosylation of the hinge region of IgA1 plays a key role in the pathogenesis of IgAN [2,3]. Human serum IgA contains two subclasses: IgA1 and IgA2; IgA1 is selectively deposited in the glomeruli of IgAN patients. Galactose-deficient IgA1 (Gd-IgA1), which lacks galactose in the hinge region of IgA1 and exposes N-acetylgalactosamine, is increased in the serum of IgAN patients and specifically deposited in the renal glomeruli of these patients [3,4,5,6].

**Figure 1:**
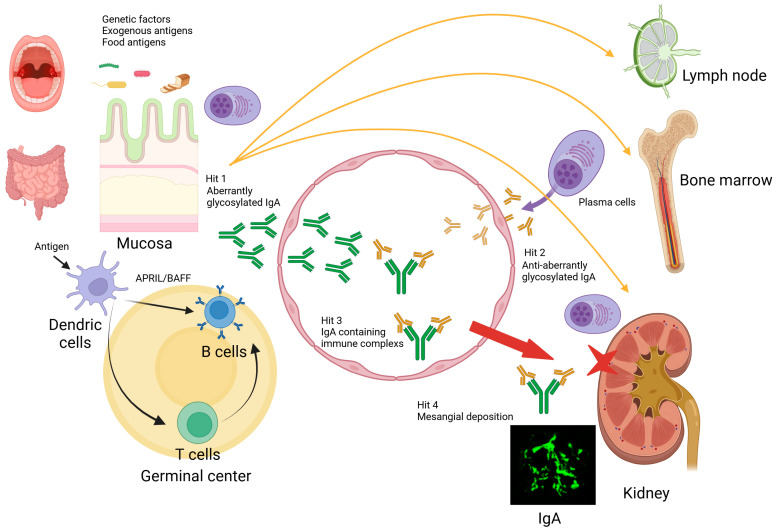
Multi-hit model of IgA nephropathy development. Hit 1: Patients and mice with IgAN have genetically increased serum levels of aberrantly glycosylated IgA. Additionally, the disruption of the mucosal immune response to exogenous antigens in mucosa-associated lymphoid tissue leads to increased levels of aberrantly glycosylated IgA via Toll-like receptors. Hits 2 and 3: autoantibodies that recognize this aberrantly glycosylated IgA develop and form nephritogenic immune complexes (ICs). Hit 4: These nephritogenic IgA-containing ICs undergo glomerular deposition. They activate mesangial cells and the complement pathway, leading to extracellular matrix proliferation, cytokine and chemokine secretion, and glomerular injury. Created with BioRender.com (accessed on 25 August 2024).

However, some IgAN patients do not exhibit elevated serum Gd-IgA1, and some patients display elevated Gd-IgA1 without nephritis [7]. Indeed, immune complexes (ICs) containing Gd-IgA1 can induce mesangial cell proliferation; this induction was not achieved using Gd-IgA1 alone [8]. Therefore, a multi-hit hypothesis was proposed to explain the pathogenesis of IgAN (Figure 1). The serum levels of Gd-IgA1 are elevated in IgAN patients (hit 1). However, elevated Gd-IgA1 alone is insufficient to cause renal damage. Aberrant glycosylated IgA1 is bound by autoantibodies to Gd-IgA1 (hit 2) and forms high-molecular-weight ICs (hit 3), resulting in mesangial deposition (hit 4) [9]. These nephritogenic IgA-containing ICs activate mesangial cells, induce extracellular matrix proliferation and the secretion of cytokines and chemokines, and cause glomerular injury [10].

Because the analyses solely based on human clinical specimens had limited success in elucidating the etiology of IgAN, disease-specific animal models are valuable tools for studying IgAN pathogenesis. However, the development of IgAN models has been challenging because of the substantial differences between humans and animals. For example, the animal model of IgAN lacks the IgA subclasses that are present in humans. Additionally, although it has N-glycans, it lacks the O-glycans of IgA, which play an important role in the pathogenesis of IgAN. Therefore, there are limitations that mean the findings of IgAN in animal models cannot be directly applied to human IgAN. Despite these differences between human and animal models, extensive research efforts have led to the development of several models with potential applications in studies regarding various specific aspects of IgAN; indeed, they have helped to elucidate IgAN pathogenesis.

## 2. Animal Models

This review presents several animal models that have contributed to the elucidation of IgAN pathogenesis and focus on the findings from these animal models (Table 1).

### 2.1. Spontaneous Mouse Models

#### 2.1.1. ddY

ddY mice derived from non-inbred dd-stock mice brought from Germany before 1920 and then raised in Japan. Imai et al. first reported that the ddY mouse, which shows the mesangial deposition of IgA and proliferation of mesangial cells during aging, is regarded as a model of spontaneous IgAN [11]. Some ddY mice exhibit high serum IgA levels at an early age and increased levels of polyclonal IgA and IgG2a, along with aging-related proteinuria. In addition, electron microscope findings also show that there were abundant electron-dense substances in the mesangial area. Thus, these mice are considered a suitable spontaneous model for the observation of the disease. However, the principal disadvantage of the ddY mouse model is that as this strain is maintained as an outbred stock, there is a considerable degree of variability in the age of onset and the severity of the disease [23].

#### 2.1.2. High IgA (HIGA)

ddY mice with high serum IgA levels at ≥ 12 weeks of age were selected and interbred 20 times to establish a strain with elevated serum IgA levels at ~25 weeks of age; this model was named the high IgA (HIGA) mouse [12]. One study revealed that renal IgA was uniformly deposited beginning at the age of ~10 weeks; light microscopy analysis of renal IgA showed a substantial increase in the mesangial matrix at an early age [24]. Furthermore, the levels of polymeric IgA were increased in the serum and renal glomeruli of HIGA mice. The kinetics of these IgA molecules indicate that IgA secretion is decreased in the intestine despite an increase in IgA + B cells, suggesting that an abnormal secretory mechanism in the intestine is responsible for the increase in the levels of serum IgA (especially polymeric IgA) [25]. Notably, the serum IgA levels in HIGA mice did not contribute to the disease onset, glomerular severity, or urinary protein. 

#### 2.1.3. Grouped ddY (gddY)

An analysis of serial renal biopsy specimens revealed that ddY mice could be classified into early-onset, late-onset, and quiescent groups [26]. The early-onset group was bred for >20 generations to establish the “100% IgAN-onset ddY” (grouped ddY (gddY)) mouse [13]. All gddY mice developed IgAN with mesangial co-deposition of IgA, IgG, and C3; mesangial proliferation; extracellular matrix expansion; and severe proteinuria by 8 weeks of age. Additionally, electron microscopy revealed high-electron-density deposition, mainly in the paramesangial area [12]. Furthermore, genomic association analysis of pathogenesis-related genes in the early-onset and quiescent groups revealed candidate genes (e.g., D10Mit86) similar to the genes identified in studies of human familial IgAN (*IGAN1*) [26,27,28]. Thus, at least part of the IgAN phenotype in this mouse model is genetically regulated in a manner similar to human disease, confirming the utility of the model.

### 2.2. Antigen Stimulation Models

In human IgAN, urinary findings are exacerbated following a respiratory tract infection and enteritis; thus, the models of IgAN caused by oral and respiratory tract antigen stimulation have been tried for a long time. Coppo et al. found that gluten and gliadin led to mesangial IgA deposition and increased serum IgA levels in BALB/c mice [14]. Additionally, the long-term exposure of mice to trichothecene vomitoxin, a naturally occurring fungal contaminant of cereal grains, caused serum IgA, IgA-containing ICs, and mesangial IgA deposition [15].

Moreover, Chintalacharuvu et al. reported that IgAN can be induced by oral immunization and a nasal challenge with the Sendai virus, similar to human respiratory viruses [16]. It is noteworthy that these experimental conditions closely resemble those of acute infection with respiratory viruses, and this model may prove invaluable for evaluating the infection-related aspects of IgAN.

### 2.3. Genetically Modified Models

The objective of using genetically modified mice is to investigate the function of specific genes and their involvement in the biological processes associated with IgAN. Various animal models were used to elucidate underlying disease mechanisms and to identify potential therapeutic targets.

Aberrantly glycosylated IgA plays a crucial role in the development of IgAN. Mice deficient in β-1,4 galactosyltransferase (β4GalT)-I, which transfers galactose from the β-1,4 linkage to terminal *N*- and *O*-linked glycans, spontaneously develop human IgAN-like glomerulitis [21]. These knockout mice exhibit an elevated proportion of high-molecular-weight IgA and a markedly elevated serum IgA concentration. Additionally, transgenic mice with serum IgA from human B-cell lymphoma 2 (Bcl-2), which regulates cell death, contain increased levels of aberrantly glycosylated IgA and show an enhanced glomerular deposition capacity compared with control mice [18]. Mice overexpressing BAFF, a factor important for the survival and differentiation of B cells, develop IgA-associated nephropathy with aberrantly glycosylated IgA [20].

FcαR (CD89), which is expressed by blood myeloid cells on mesangial cells, is thought to function in the pathogenesis of IgAN. Because mice lack a homologous molecule for human CD89, transgenic mice were created that express human CD89 and show mesangial IgA deposition, macrophage infiltration of the glomerulus and interstitium, expansion of the mesangial matrix, hematuria, and mild proteinuria [17]. Because mice also lack the IgA subclass, a humanized mouse model was established with IgA1 knock-in that is also human CD89 transgenic: the α1KICD89Tg mouse [22].

Wang et al. previously demonstrated that the overexpression of LIGHT, a ligand expressed on activated T cells, results in the induction of splenic chemokine production and lymphoid tissue formation via the lymphotoxin β receptor (LTβR). The interaction between LIGHT and LTβR stimulates the production of IgA in the intestine while simultaneously impairing IgA transport within the intestinal lumen. These changes result in a significant increase in serum high-molecular-weight IgA and the glomerular deposition of IgA [19].

## 3. A Multi-Hit Hypothesis

### 3.1. Nephritogenic IgA

Aberrantly glycosylated IgA1 has a self-aggregation capacity and is recognized by autoantibodies, facilitating IC formation. Although the hinge region of human IgA1 contains three to five *O*-linked glycans, murine IgA has *N*-glycans but lacks *O*-glycans. Notably, mice deficient in β4GalT-I, which transfers galactose from the β-1,4 linkage to terminal *N*- and *O*-linked glycans, spontaneously develop human IgAN-like glomerulitis [21]. Higher serum IgA levels and increased accumulation of polymeric IgA were observed in β4GalT-I-deficient mice [21]. Additionally, the overexpression of Bcl-2 in B cells leads to the dysregulation of B-cell apoptosis and enhances systemic IgA immune responses [18]. Serum IgA from human Bcl-2 transgenic mice contains increased levels of aberrantly glycosylated IgA and displays enhanced glomerular deposition capacity compared with serum IgA from control mice [18]. These findings suggest that despite their deficiency in *O*-glycan, mouse models can serve as a useful tool for the analysis of nephritogenic IgA.

Biotinylated *Ricinus communis* agglutinin (RCA-I) and *Sambucus nigra* bark lectin (SNA) can measure aberrant glycosylation of IgA in a lectin-binding assay. Because RCA-I and SNA recognize terminal galactose and terminal sialic acid, respectively, decreased binding of RCA-I and SNA indicates an increase in aberrantly glycosylated IgA (with fewer terminal galactoses and sialic acids, respectively). This lectin-binding assay was used to analyze the glycosylation of IgA in gddY, HIGA, and BALB/c mice. The results show that serum IgA from gddY mice had significantly weaker lectin SNA binding compared with serum IgA from HIGA and BALB/c mice, as well as significantly weaker RCA-I binding compared with BALB/c mice; these findings indicate that gddY mice have increased levels of aberrantly glycosylated IgA with few terminal sialic acids and/or galactoses. Furthermore, the levels of serum and glomerular IgA–IgG2a ICs, as well as proteinuria, were substantially increased in the gddY mice compared with HIGA mice [29]. These findings suggest that aberrantly glycosylated IgA in gddY mice forms ICs and glomerular deposits, leading to renal injury. 

Recently, Nihei et al. reported IgA-type autoantibodies against β2-spectrin expressed on the surface of mesangial cells in serum from gddY mice and patients with IgAN, considerably advancing the broader understanding of IgAN pathogenesis [30]. The relationship between this autoantibody and aberrantly glycosylated IgA is currently under investigation.

In humans, IgA has two subclasses, IgA1 and IgA2, and IgA1 shows an *O*-glycan in the hinge region. IgA extracted from the renal glomeruli of patients with IgAN was reported to be predominantly IgA1, with significantly reduced *O*-glycan side chain glycosylation in the hinge region [4,5]. This demonstrates that reduced galactosylation and oversialylation of the IgA1 hinge glycopeptide are pivotal to the glomerular deposition of IgA. This is further supported by the results of experiments using KM55, an anti-Gd-IgA1 monoclonal antibody that we recently established [3]. The human α1 heavy chain contains multiple O-linked glycan chains, which are attached to serine and threonine residues in the hinge region, specifically at positions 3–6 [31,32].

### 3.2. The Role of IgA-Containing ICs in the Pathogenesis of IgAN

To investigate the role of IgA-containing ICs in the pathogenesis of IgAN, a model of IgA onset and progression was established using IgA-containing ICs. In 1979, Rifai et al. successfully established an IgAN model mouse using ICs that contained IgA antibodies to dinitrophenyl and bovine serum albumin-bound dinitrophenyl [33]. They showed that polymeric IgA, but not monomeric IgA, was critical for the glomerular deposition of the complexes and induction of nephritogenic histological changes. Furthermore, they demonstrated that only large ICs, where the IC is coupled with the polymeric form of IgA, are deposited in glomeruli. Additionally, single-dose injection experiments with ICs showed that IC deposition in glomeruli disappears after a short period of time, indicating that persistent IC deposition in mesangial lesions requires a continuous supply of serum ICs [34,35].

**Figure 2:**
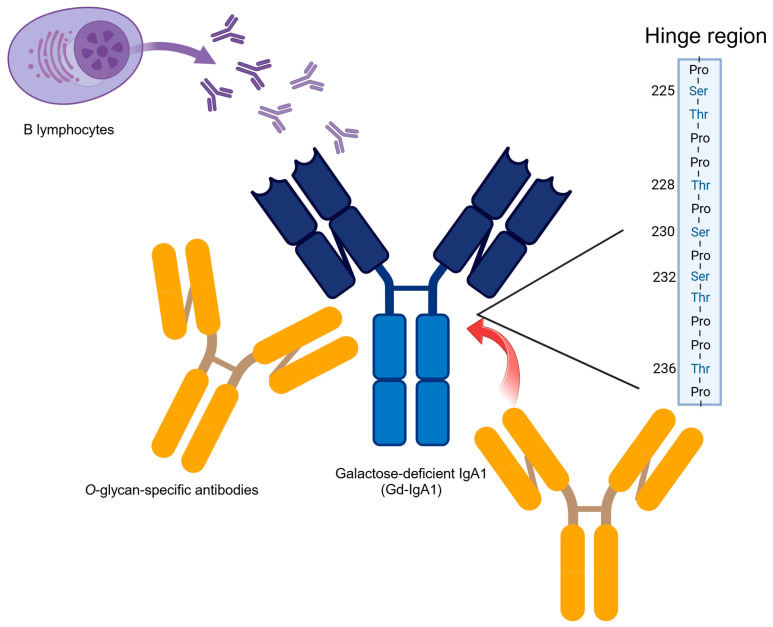
Mechanism of galactose-deficient IgA1 (Gd-IgA1) and immunological complexes (ICs) formation in humans. The human α1 heavy chain contains several *O*-linked glycan chains at positions 3–6, attached to the Serine and Threonine residues in the hinge region. B lymphocytes produce IgA1 with a galactose deficiency in the hinge region of the heavy chain. IgG antibodies recognize GalNAc-containing epitopes on the galactose-deficient hinge region *O*-glycans of IgA1, resulting in the formation of ICs. Created with BioRender.com (accessed on 25 August 2024). Thr: Threonine, Ser: Serine, Pro: Proline.

In humans, Yanagihara et al. stimulated human mesangial cells with IgA1 extracted from patients with IgA myeloma and discovered that only polymeric IgA1 or ICs, but not monomeric IgA1, activated mesangial cells [36]. These findings suggest that the formation of ICs induced by Gd-IgA1 may contribute to the exacerbation of IgAN. Nevertheless, the production mechanism of Gd-IgA1-specific IgG/IgA antibodies and the involvement of autoantibodies against Gd-IgA1 in the formation of pathogenic ICs remain unclear. Tomana et al. previously demonstrated that removing *O*-glycans from the hinge region of IgA1 significantly reduced the reactivity of serum IgG extracted from patients with IgAN to IgA1 [37]. Suzuki et al. generated IgG-secreting cells immortalized with Epstein–Barr virus from patients with IgAN and showed that the secreted IgG formed glycan-dependent ICs with Gd-IgA1 [9]. In a recent study, Rizk et al. demonstrated the presence of IgG in the glomerular deposits of patients with IgAN. This IgG showed specificity for Gd-IgA1, including IgG that could not be detected by conventional immunofluorescence microscopy [38]. These findings provide further support for the hypothesis that Gd-IgA1-specific IgG autoantibodies play an important role in IgAN pathogenesis (Figure 2). 

In mice, the mechanism by which these pathogenic ICs deposit in the glomerulus, activate mesangial cells, and cause glomerular injury was studied. When nude mice were injected with aberrantly glycosylated IgA from gddY mice, IgA deposition along the glomerular capillary wall occurred in a focal and segmental manner, with acute cellular activation [39]. A complex consisting of purified high-molecular-weight human Gd-IgA1 myeloma protein and IgG isolated from the sera of IgAN patients (engineered IC: EIC) was injected into the nude mice; the complex demonstrated co-deposition with mouse C3 in the glomerular mesangium, which resulted in glomerular injury. In contrast, IgG isolated from the serum of three healthy volunteers did not show deposition with Gd-IgA1. Additionally, IgA1 alone (in either the polymeric or monomeric form) was not detected in the glomeruli, and no deposition of C3 was observed [40]. We have reported that glomerular immune deposits and pathological changes similar to those of human IgAN were induced in nude mice through the injection of in vitro-formed human Gd-IgA1-IgG ICs [41]. Gd-IgA1-containing ICs stimulate glomerular endothelial cells, causing the activation of inflammatory cytokines, chemokines, and adhesion molecules. This process results in endothelial damage and altered endothelial permeability, allowing ICs to migrate into the mesangium [41]. These findings indicate that in IgAN patients, Gd-IgA1-containing ICs undergo mesangial deposition through a balance of supply and removal in the glomerular region, activating mesangial cells and inducing glomerular injury.

## 4. The Role of Key Molecules

### 4.1. IgA Receptors

Soluble CD89-IgA complexes were detected in the serum of IgAN patients, highlighting the involvement of IgA receptors in the etiology of IgAN. However, whereas humans have two IgA isotypes, mice have only one IgA isotype. Additionally, unlike human IgA1, mouse IgA has a short hinge region that does not contain an *O*-glycan. Therefore, Montello et al. crossed human IgA1 knock-in mice with human CD89 transgenic mice to create a humanized mouse model: the α1KICD89Tg mouse [22]. Histological examination revealed mesangial IgA1 and C3 deposition, glomerular macrophage infiltration, and mesangial cell proliferation.

α1KICD89Tg mice also show an increased expression of transferrin receptor 1 (CD71), which is the major IgA1 receptor on the mesangial cell surface in IgAN patients and on the intestinal cell surface in celiac disease patients. In the α1KICD89Tg mouse model, the overexpression of transglutaminase 2 and CD71 causes mesangial deposition of the IgA1 complex [22].

Celiac disease is a systemic disease caused by serum anti-tissue transglutaminase 2 antibodies and anti-gliadin antibodies due to gluten, which primarily affects the small intestine. An association between celiac disease and IgAN has been suggested, especially in Europe. Since the 1990s, the role of gluten in the physiopathology of IgAN has been supported by uncontrolled studies that detected anti-gliadin antibodies, along with a gluten-free diet that resulted in decreased proteinuria [42]. Additionally, α1KICD89Tg mice fed a gluten-free diet for three generations showed substantial reductions in mesangial IgA1 deposition and hematuria, along with a loss of the IgA1-soluble CD89 (sCD89) complex, and decreases in the mesangial expression of CD71 and transglutaminase 2 [43].

Furthermore, reintroduction of the gluten diet caused disease recurrence, including an increase in the serum level of IgA1 anti-gliadin antibodies, which was correlated with urinary protein. Intestinal IgA1 secretion was also increased; intestinal inflammation and villous atrophy were exacerbated. These findings suggest a mechanism that directly involves the induction of IgA anti-gliadin complexes by the food antigen gliadin and subsequent interactions with circulating sCD89, at least in these mice [43].

### 4.2. Dysregulation of Mucosal Imunity

IgAN is exacerbated by mucosal infections, especially upper respiratory tract infections, suggesting that the dysregulation of the mucosal immune system is involved in this disease [44]. Notably, gddY mice did not develop IgAN in germ-free conditions; nephritis could be reconstituted by transitioning those mice from germ-free conditions to specific pathogen-free conditions [45]. These facts suggest that the microbiota is important for the development of IgAN. Recently, Higashiyama et al. reported that oral bacteria induce IgA autoantibodies against a mesangial protein in gddY mouse [46]. In humans, there have been reports that the salivary and gut microbiota of IgAN patients differed from that of healthy controls [47,48,49]. Thus, particular strains of commensal bacteria may induce an immune response that leads to the production of nephritogenic IgA. Therefore, animal models of IgAN based on oral and trans-respiratory antigen stimulation have been attempted for many years.

In 1983, Emancipator et al. demonstrated that orally administered antigens induced specific mucosal IgA, resulting in the glomerular mesangial deposition of ICs containing specific IgA antibodies [50]. They showed that IgA deposits contained J chains, indicating that at least some deposition in the kidney involves dimeric or polymeric IgA [50]. Coppo et al. subsequently demonstrated that the oral administration of gluten to BALB/c mice can induce IgAN by increasing the serum IgA antibodies to gliadin and the deposition of those antibodies in renal glomeruli [14]. As a model for the induction of this disease by oral immunomodulators, Pestka et al. administered a diet containing vomitoxin (i.e., deoxynivalenol) to mice; this diet induced significant increases in serum IgA, IgA-containing ICs, and mesangial IgA deposition with hematuria in a manner similar to the manifestations of human IgAN [15]. Deoxynivalenol has been demonstrated to increase IL-6 expression in vivo and in vitro, which are associated with increased IgA production. IL-6 is crucial for mucosal IgA immunity, both due to its impact on the differentiation of IgA-producing B cells and its stimulation of production by macrophages, T cells, and other cells in the gut [51,52,53]. Consequently, it is postulated that deoxynivalenol facilitates the differentiation of IgA-secreting cells through its interaction with CD4 + T cells and other cells in the gut.

Wang et al. reported that intestinal inflammation caused by the overexpression of LIGHT induces splenic chemokine production and lymphoid tissue formation via LTβR [19]. The LIGHT–LTβR interaction stimulates IgA overproduction in the intestine while causing impaired IgA transport in the intestinal lumen; these changes lead to dramatic increases in serum high-molecular-weight IgA and the renal deposition of IgA [19]. Therefore, the dysregulation of the LIGHT–LTβR pathway presumably causes intestinal inflammation and increased IgA synthesis; it may contribute to the onset of murine IgAN.

An IgAN-like mouse model was reportedly induced by antigen stimulation of the respiratory tract and by oral challenge. Intranasal exposure to the Sendai virus can induce IgAN-like renal injury in mice by inactivating T-helper 2 cells [16]. Importantly, because these experimental conditions mimic acute exposure to respiratory viruses, the Sendai virus exposure model is useful for elucidating the pathogenesis associated with an upper respiratory tract infection among IgAN patients.

Overall, these experimental results in animal models support the notion that mucosal exposure to food and microbial antigens induces the mucosal macromolecules IgA and IgA-containing ICs, leading to IgAN-like injury in glomeruli.

### 4.3. Toll-like Receptors (TLRs)

Certain exogenous microbial antigens (e.g., *Haemophilus parainfluenzae* and methicillin-resistant *Staphylococcus aureus*) have been identified in the kidneys of IgAN patients [54,55]. It was suggested that this specific antigen and IgA form an IC on mucosal surfaces, in circulation, or locally within the kidney; these IgA-containing ICs may induce nephritis [56]. However, there is no evidence that IgAN patients exhibit renal glomerular deposition of specific exogenous antigens. 

Therefore, a potential role for TLRs in the pathogenesis of IgAN was proposed. TLRs are central molecules of the innate immune system that act as anti-pathogen defense mechanisms by recognizing antigenic structures common to bacteria and viruses. Regardless of the specific antigens, overactivation of the innate immune system may lead to the development or progression of IgAN through the increased production of nephritogenic IgA and ICs. Myeloid differentiation factor 88 (MyD88) is an important TLR signaling molecule involved in the innate immune function. A genetic analysis of gddY mice revealed that MyD88 is strongly associated with the development of IgAN. Additionally, an enhanced expression of TLR9, which recognizes bacterial and viral unmethylated DNA (CpG DNA), was observed in the spleens of gddY mice. Intranasal administration of synthetic CpG DNA to gddY mice increased the serum levels of IgA and ICs; it also exacerbated nephritis, with increases in glomerular mesangial IgA deposition and urinary protein excretion [57].

Recently, Lee et al. showed that both DNA-sensing TLR9 and RNA-sensing TLR7 contribute to IgAN progression through the production of aberrantly glycosylated IgA [58]. Although the cytokine pattern varies according to ligand type, the downstream pathways of TLR9 and TLR7 share many common factors. The MyD88-specific nuclear factor κB (NF-κB) pathway, downstream of TLR7/9, triggers the release of inflammatory cytokines (e.g., interleukin-6 [IL-6]) and is involved in the production of aberrantly glycosylated IgA [58,59]. Additionally, Lee et al. demonstrated that hydroxychloroquine, a therapeutic agent for malaria and autoimmune diseases, suppresses the production of aberrantly glycosylated IgA and ICs by inhibiting TLR7/9 signaling and the activities of downstream cytokines [58]. Indeed, several clinical trials recently showed that hydroxychloroquine can reduce proteinuria in IgAN patients [60]. These findings indicate that the regulation of nucleotide-sensing TLRs is a potential therapeutic strategy for IgAN.

### 4.4. A Proliferation-Inducing Ligand (APRIL)

APRIL and BAFF are type 2 transmembrane proteins within the tumor necrosis factor ligand superfamily. These two proteins—produced by immune cells, such as monocytes and dendritic cells—regulate B-cell differentiation and survival via binding to receptors on the B-cell surface [61,62].

In studies of gddY mice, we found that the TLR9/TLR7 MyD88–NF-κB pathway induces pro-inflammatory cytokines, including IL-6; upregulates APRIL; and promotes the synthesis of aberrant glycosylated IgA [58,59]. Furthermore, we found that an anti-APRIL antibody treatment suppressed the serum levels of IgA and aberrantly glycosylated IgA, reduced the deposition of mesangial IgA and IgG, and decreased the excretion of urinary protein [63,64]. This anti-APRIL antibody treatment also reduced the numbers of monkey IgA-positive mononuclear cells in the lymphoid tissues of cynomolgus monkeys, which share more than 99% amino acid sequence identity with human APRIL [63].

A genome-wide association study indicated that *TNFSF13*, which encodes APRIL, may contribute to the development of human IgAN. Moreover, correlations were observed between the elevated serum levels of APRIL and the prognosis of IgAN patients, suggesting that IgAN progression is related to APRIL activation [20,65,66]. We previously reported that APRIL is overexpressed in tonsillar germinal centers among IgAN patients compared with chronic tonsillitis patients; B cells in these germinal centers exhibited particularly high expressions of APRIL. Additionally, the proportion of APRIL-positive cells was correlated with the amount of urinary protein and the extent of serum Gd-IgA1 reduction after a tonsillectomy [67]. These findings suggest that the mucosal activation of APRIL plays an important role in the development and progression of IgAN.

The above observations prompted the recent initiation of clinical trials using anti-APRIL antibodies. A phase II multicenter, double-blind, randomized, placebo-controlled study showed that 12 months of treatment with sibeprenlimab, an anti-APRIL antibody, significantly reduced the proteinuria compared with a placebo [68]. Based on these results, two phase III multicenter, double-blind, randomized, placebo-controlled clinical trials of anti-APRIL antibody treatment for IgAN patients are currently underway, with the potential to establish a novel approach for IgAN management.

Concurrently, an association between BAFF and IgAN pathogenesis was suggested. One report has shown that mononuclear cells from the tonsils of IgAN patients overproduced BAFF and IgA upon stimulation with CpG oligodeoxynucleotides, which bind to TLR9. BAFF expression in tonsil cells showed a greater increase among IgAN patients than among non-IgAN patients [69]. These findings suggest that tonsil cells are affected by BAFF molecules and contribute to the development of IgAN. Although BAFF-overexpressing mice develop IgA-associated nephropathy, this nephropathy does not develop under germ-free conditions [20]. When these germ-free mice were colonized by a limited set of commensal microbiota (i.e., Altered Schaedler Flora), IgA-associated nephropathy re-emerged, along with the appearance of specific antibodies in the circulation [20]. However, BAFF-overexpressing mice represent a particularly artificial model because their serum IgA levels are abnormally elevated (50–100-fold higher than the levels in normal mice). Although glomerular IgA deposition was observed in the kidneys of these mice, the microscopic findings were partially inconsistent with the typical characteristics of IgAN, such as the presence of an excessive mesangial matrix. There have been few reports of a prognostic association between BAFF and IgAN; notably, the BRILLIANT clinical trial, which was a randomized, double-blind, placebo-controlled phase II/III study, of a soluble BAFF inhibitor (belimumab) was discontinued. Thus, further research is needed to investigate the potential association between BAFF and IgAN.

### 4.5. Mucosa-Bone Marrow Axis in IgAN

Reports have shown that IgAN remission could be achieved through allogeneic peripheral blood stem cell transplantation in acute lymphocytic leukemia patients [70,71]. Additionally, abnormal bone marrow cell composition was observed in IgAN patients. Imasawa et al. demonstrated that bone marrow transplantation from HIGA mice into normal mice could reproduce IgAN [72]. We performed further investigation of this bone marrow abnormality in gddY mice. Our results show that the transplantation of bone marrow from gddY mice into normal control mice could reproduce IgAN, whereas the transplantation of bone marrow from normal mice into gddY mice ameliorated IgAN while reducing glomerular IgA deposition [73]. These findings suggest that cells responsible for the pathogenesis of murine IgAN are located in the bone marrow. Furthermore, bone marrow transplantation from normal mice resulted in the loss of glomerular IgA, indicating that the nephritogenic IgA involved in pathogenesis may be continuously supplied to the glomeruli and rapidly cleared. Next, for the determination of whether responsible cells in the bone marrow require extramedullary homing to produce nephritogenic IgA, we used alymphoplasia mice (aly/aly mice) that were congenitally deficient in secondary lymph nodes (e.g., Peyer’s patches) and serum IgA. The transplantation of bone marrow from gddY mice into aly/aly mice revealed similar glomerular IgA deposition, confirming that the responsible bone marrow-derived cells produced nephritogenic IgA without additional homing to the intestinal mucosa or secondary lymph nodes [74]. Although this finding suggests that nephritogenic IgA is produced in the bone marrow, it also emphasizes the importance of secondary lymph nodes in the development of nephritis after IgA deposition—transplanted aly/aly mice showed glomerular IgA deposition without developing substantial glomerular damage. Finally, to investigate whether the responsible cells originated from lymphoid tissues other than bone marrow, we transferred splenocytes from gddY mice into normal mice; surprisingly, this transfer led to the reconstitution of IgAN [75]. These findings indicate that the responsible cells had spread into systemic lymphoid tissues.

In a recent study, BAFF transgenic mice exposed to *Neisseria* nasal infection exhibited significantly elevated serum anti-*Neisseria* IgA levels and an increased level of anti-*Neisseria* IgA-secreting cells within the kidneys. Additionally, a study using renal biopsy samples from IgAN patients revealed the presence of CD19-positive B cells in renal tissue; these cells were co-localized with IgA deposits [76]. Currie et al. found an increased prevalence of *Neisseria* genus carriage in the tonsils, as well as an increase in serum anti-*Neisseria* IgA, among IgAN patients [77]. These results suggest that exogenous airway exposure causes IgA-producing cells to migrate into renal tissue, where they contribute to the pathogenesis of IgAN. However, further studies are needed to elucidate the mechanisms by which these cells migrate from the airways to the kidneys, as well as the role of IgA-producing cells within the kidneys, in the pathogenesis of IgAN.

### 4.6. Endothelin

Endothelin-1 (ET-1), a potent vasoconstrictor mainly produced by vascular endothelial cells, plays important roles in inflammation, tissue injury, and remodeling [78]. ET receptors constitute two types of G protein-coupled receptors: endothelin A (ETA) and endothelin B (ETB). Within renal glomeruli, ET-1 predominantly binds to ETA receptors in afferent arterioles and ETB receptors in efferent arterioles; it also binds to ETA receptors in podocytes and mesangial cells. ET-1 promotes cell proliferation and extracellular matrix production in mesangial cells via the ETA receptor; moreover, ET-1 is involved in the pathogenesis of glomerulosclerosis. In podocytes, proteinuria is suspected to develop through the decreased expression of slit membrane proteins, loss of foot processes, and induction of apoptosis.

A report has shown that ET-1 and ETA receptor expression levels were correlated with the progression of renal damage in ddY mice; treatment with ETA receptor antagonists reduced the urinary protein levels and prevented decreases in the estimated glomerular filtration rate [79]. Recently, Nagasawa et al. showed that the administration of sparsentan, a dual antagonist of endothelin angiotensin receptors, suppressed urinary protein excretion and glomerulosclerosis in gddY mice compared with control mice [80]. Furthermore, sparsentan treatment prevented the loss of podocytes and glomerular glycocalyx, while suppressing the expression of inflammatory markers, such as NF-κB and IL-6, as well as fibrotic markers (e.g., transforming growth factor-β). Compared with losartan, a typical angiotensin receptor blocker, under equivalent systolic blood pressure-lowering conditions, sparsentan demonstrated a more rapid antiproteinuric effect that preserved the numbers of podocytes and extent of glomerular glycocalyx, thereby inhibiting glomerulosclerosis [80].

Glycocalyx covers the luminal surface of vascular endothelial cells and prevents the free passage of proteins and inflammatory cells. The loss of glomerular glycocalyx is associated with increased vascular permeability and participates in renal injury. Recently, we reported that Gd-IgA1-IgG ICs induce glomerular filtration barrier dysfunction via glycocalyx loss in endothelial cells, promote the migration of ICs to mesangial regions, and trigger the activation of inflammatory cytokines and chemokines. These findings suggest that sparsentan exerts a protective effect on endothelial cells, thereby inhibiting IC migration into the mesangium and suppressing the subsequent inflammatory response.

Additionally, other recent studies showed that EIC-induced glomerular hypercellularity was inhibited by the oral administration of sparsentan to mice intravenously injected with EICs mimicking human IgAN [81]. Comprehensive analysis of the renal transcriptome revealed that key inflammatory and proliferative biological genes and pathways (e.g., members of the MAP kinase family, integrin components, complement genes, and Fc receptor elements) with elevated expression in this EIC model of IgAN were significantly suppressed by sparsentan [81]. Similar inflammation-related pathways were identified in the single-cell analyses of kidneys from gddY mice, ref. [82] suggesting that sparsentan protects against renal injury by suppressing inflammatory pathways.

Increased urinary protein levels are observed in cases of human IgAN with elevated glomerular ET-1 expression, suggesting that the endothelin system plays a central role in IgAN progression [83]. Indeed, the ongoing phase III PROTECT, a double-blind, randomized, active-controlled trial, showed that compared with the angiotensin receptor blocker irbesartan, sparsentan exhibited superior renal function preservation and antiproteinuric effects in IgAN patients [84,85]. Although side effects, such as fluid retention, should be carefully monitored, ETA receptor antagonists represent promising new treatments for IgAN.

## 5. Conclusions

Because mouse models of IgAN lack *O*-glycans in IgA, the findings in murine IgAN cannot be directly applied to studies of human IgAN. However, dynamic experiments, such as stimulation, loading, bone marrow transplantation, and genetic modification, cannot be performed in humans; thus, animal experiments are essential. Several mouse models of IgAN have been established, helping to elucidate various aspects of IgAN pathogenesis and facilitate the development of IgAN-specific drugs. We expect that further elucidation of IgAN pathogenesis and the development of novel therapies using appropriate experimental models will continue to improve the prognosis of IgAN patients.

## Figures and Tables

**Table 1:** Summary of selected IgA nephropathy (IgAN) mouse models.

Name	Year	Features
**Spontaneous models**		
ddY	1986	Spontaneous model with mesangial deposition of IgA and aging-related proliferation of mesangial cells [11].
High IgA	1997	ddY mice with high serum IgA levels were selected and interbred more than 20 times [12].
Grouped ddY	2012	Established by interbreeding of the early-onset group for > 20 generations. gddY mice exhibit glomerular lesions characterized by mesangial proliferation with co-deposition of IgA, IgG, and C3 [13].
**Antigen stimulation models**		
Gluten	1989	Gluten induces the renal deposition of IgA in BALB/c mice, simultaneously increasing serum levels of anti-gliadin IgA [14].
Vomitoxin	1989	Administration of a diet containing vomitoxin (deoxynivalenol) to mice significantly increased serum IgA, IgA-containing ICs, and mesangial IgA deposition with hematuria [15].
Sendai virus	2001	Intranasal exposure to the Sendai virus induced IgAN-like renal injury by inactivating T-helper 2 cells [16].
**Genetically modified models**		
CD89	2000	CD89 transgenic mice with circulating CD89–IgA complex developed spontaneous massive mesangial deposition of IgA and expansion of mesangial matrix [17].
Human Bcl-2	2001	Human Bcl-2 transgenic mice exhibited increased levels of aberrantly glycosylated IgA and increased glomerular deposition of IgA [18].
LIGHT	2004	Dysregulated LIGHT expression on T cells caused impaired IgA transport in the intestinal lumen, resulting in increased serum levels of polymeric IgA and increased renal deposition of IgA [19].
BAFF	2006	BAFF-overexpressing mice developed IgA-associated nephropathy with increased serum levels of commensal-dependent bacteria-reactive IgA [20].
β1,4-galactosyl-transferase-I-deficient	2007	Mice deficient in β-1,4 galactosyltransferase-I spontaneously developed human IgAN-like glomerulitis with elevated serum levels of IgA and the accumulation of polymeric IgA [21].
α1KICD89Tg	2012	α1KI-CD89Tg mice showed mesangial deposition of IgA1 with glomerular macrophage infiltration and mesangial cell proliferation, resulting in proteinuria and hematuria [22].

Bcl-2: B-cell/CLL lymphoma 2, BAFF: B-cell activating factor.
